# Epidemiology of Dengue — Puerto Rico, 2010–2024

**DOI:** 10.15585/mmwr.mm7349a1

**Published:** 2024-12-12

**Authors:** Dania M. Rodriguez, Zachary J. Madewell, Jomil M. Torres, Aidsa Rivera, Joshua M. Wong, Gilberto A. Santiago, Vanessa Rivera-Amill, Gabriela Paz-Bailey, Melissa Marzan-Rodriguez, Laura E. Adams

**Affiliations:** ^1^Dengue Branch, Division of Vector-Borne Diseases, CDC; ^2^Puerto Rico Department of Health; ^3^Ponce Health Sciences University/Ponce Research Institute, Ponce, Puerto Rico.

**Summary**

**What is already known about this topic?**

Cases of dengue, a mosquitoborne viral illness, are increasing worldwide; during 2024, approximately 13 million cases have been reported in the Americas.

**What is added by this report?**

During 2023–2024, the median age of patients with dengue, the percentage of patients hospitalized, and the prevalences of serotypes 2 and 3 increased compared with the previous decade (2010–2019).

**What are the implications for public health practice?**

Understanding the changing epidemiology of dengue can help guide public health action, including providing clinical training, strengthening surveillance, ensuring health care system resilience, and raising public awareness.

## Abstract

Dengue is a mosquitoborne viral illness that can cause acute febrile illness, severe disease, or death. Worldwide, the number of dengue cases is increasing. During the last dengue outbreaks in Puerto Rico throughout 2010–2013, dengue virus (DENV) serotype 1 (DENV-1) predominated, and the largest proportion of cases occurred among adolescents and young adults aged 10–19 years. Dengue case data from January 1, 2010–November 4, 2024, were obtained from the Puerto Rico Department of Health. Bivariate analyses were conducted to evaluate the distribution of cases by patient age, DENV serotype, and hospitalization status during three periods: 2010–2019, 2020–2022, and 2023–2024. During 2023–2024, the median age of dengue cases increased to 26 years (95% CI = 25–27 years) compared with that during 2020–2022 (17 years; 95% CI = 17–18 years) and 2010–2019 (19 years; 95% CI = 19–19 years). After >10 years of DENV-1 predominance, the proportions of DENV serotypes 2 (DENV-2) and 3 (DENV-3) increased significantly during 2023–2024, with DENV-3 replacing DENV-1 as the predominant serotype. In addition, the proportion of dengue patients who were hospitalized increased from 35.7% (2010–2019) to 53.5% (2023–2024). The current dengue outbreak in Puerto Rico marks a shift in serotype predominance to DENV-3 and increasing percentages of cases in older age groups (61.7% in adults aged ≥20 years), although a high proportion of cases still occur among adolescents aged 10–19 years (29.5%). The current dengue outbreak also has a higher rate of hospitalizations than those in previous years. Understanding the changing epidemiology of dengue is crucial to guiding public health strategies for dengue control, including clinical management, surveillance and health care system resilience, and public outreach and education.

## Introduction

Dengue, a viral illness transmitted by mosquitoes (most commonly *Aedes aegypti* and *Aedes albopictus*), can cause asymptomatic infection, nonspecific acute febrile illness, or severe dengue, which includes severe bleeding, critical organ failure, or plasma leakage (*1**,**2*). With appropriate treatment, including early clinical and laboratory diagnosis and maintenance of adequate hydration, mortality is typically <1%.* Four distinct dengue virus (DENV) serotypes (DENV-1–4) cause illness. Infection with one serotype provides long-lasting immunity against that specific serotype, but only transient protection against the other three serotypes (*3*). Dengue prevention includes avoiding mosquito bites, using mosquito repellents, wearing protective clothing, (i.e., long sleeves and pants), and eliminating water-holding containers that can serve as mosquito breeding sites. Dengvaxia,^†^ a dengue vaccine recommended for persons aged 9–16 years with evidence of previous DENV infection and living in endemic areas, provides protection against all four DENV serotypes among persons with previous DENV infection; it is not recommended for persons without previous infection because of an increased risk for hospitalization or severe dengue. However, the vaccine is being discontinued by the manufacturer because of a lack of demand in the global market. In Puerto Rico, dengue is endemic, with outbreaks occurring every 3–7 years. This report describes dengue cases reported to the Puerto Rico Department of Health (PRDH) during January 1, 2010–November 4, 2024.

## Methods

Patients meeting the Council of State and Territorial Epidemiologists’ dengue case definition^§^ were included in the analysis. Dengue cases were aggregated by period (2010–2019, 2020–2022, and 2023–2024), patient age (<20, 20–39, and ≥40 years), identified DENV serotype, and patient hospitalization status. Periods were selected based on dengue trends, with 2010–2019 including the 2010–2013 dengue outbreaks and subsequent low-prevalence years (2014–2019); 2020–2022, including the reintroduction and increased transmission of DENV; and 2023–2024, capturing the most recent outbreak period. Differences in the median patient age by period were evaluated using Mood’s median tests. Hospitalization rates by period, serotype, and age group were evaluated using logistic regression models; adjusted odds ratios and 95% CIs are reported. Analyses were conducted using R (version 4.4.0; R Foundation). This activity was reviewed by CDC, deemed not research and was conducted consistent with applicable federal law and CDC policy.^¶^

## Results

### Dengue Case Characteristics

A total of 39,094 dengue cases were reported to PRDH during 2010–2024, including 30,517 (78.1%) during 2010–2019; 2,695 (6.9%) during 2020–2022; and 5,882 (15.0%) during 2023–2024 (Figure). Large dengue outbreaks occurred in 2010 (10,967 cases), 2012 (6,583), and 2013 (10,351), followed by an unusually low number of cases during 2016–2019 (365), likely associated with temporary crossreactive protective immunity from the Zika virus disease outbreak in 2016 (Supplementary Figure, https://stacks.cdc.gov/view/cdc/174510). The median patient age across the full study period was 20 years (range = 0–102 years). The median age during 2010–2019 was 19 years, declined to 17 years during 2020–2022, and then increased to 26 years during 2023–2024. Incidence increased among persons aged 20–39 years from 78.9 cases per 100,000 population during 2010–2019 to 101.9 during 2023–2024, and among persons aged ≥40 years, from 43.3 to 53.9 (Table 1). Fewer than one half (45.6%) of patients were female. A total of 94 (0.2%) fatal dengue cases were reported during 2010–2024.

**FIGURE:**
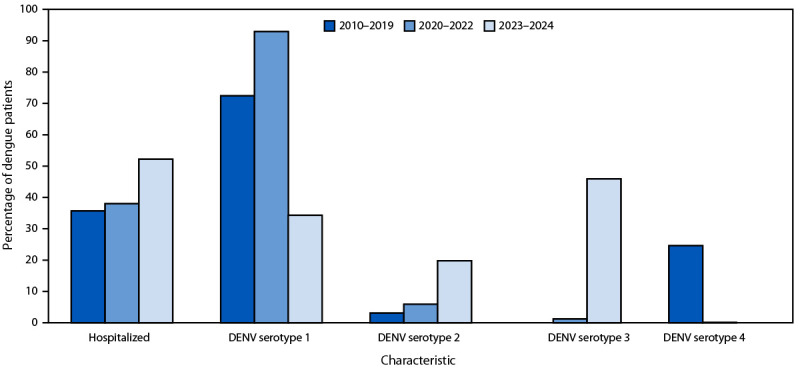
Percentage of dengue patients hospitalized and percent distribution of infecting serotype,* by surveillance period (N = 39,094) — Puerto Rico Department of Health, Puerto Rico, 2010–2024 **Abbreviation**: DENV = dengue virus. * Number of dengue cases with information on infecting serotype divided by total dengue cases: 2010–2019 = 20,783 / 30,517; 2020–2022 = 1,512 / 2,695; and 2023–2024 = 4,753 / 5,882). Percentages were calculated among patients for whom serotype was known.

**TABLE 1:** Estimated average annual dengue incidence,* by age group and period — Puerto Rico, 2010–2024

Age group, yrs	Average annual incidence*		
	2010–2019	2020–2022	2023–2024
<20	184.4	79.7	196.1
20–39	78.9	27.8	101.9
≥40	43.3	9.5	53.9

* Cases per 100,000 population, calculated using the number of dengue cases reported to the Puerto Rico Department of Health and U.S. Census Bureau data. https://www.census.gov/data.html

### Dengue Serotype Trends

Among 27,048 (69.2%) cases with an identified serotype during 2010–2024, DENV-1 accounted for two thirds (18,072; 66.8%); DENV-2, DENV-3, and DENV-4 accounted for 6.2% (1,664), 8.2% (2,206), and 18.9% (5,106) cases, respectively (Figure). During 2010–2019, DENV-1 was identified in 15,039 (72.4%) cases, with DENV-2 and DENV-4 identified in 635 (3.1%) and 5,104 (24.6%) cases, respectively. During 2020–2022, DENV-1 predominance increased to 92.9%, with low percentages of DENV-2 (5.9%) and DENV-3 (1.2%) identified. During 2023–2024, DENV-3 emerged as the predominant serotype (45.9%) accompanied by a reduction in the prevalence of DENV-1 (34.3%) and an increase in the prevalence of DENV-2 (19.8%). DENV-4 prevalence declined from 24.6% during 2010–2019, to 0.1% during 2020–2022, to 0% during 2023–2024. During these periods, no coinfections with more than one serotype were identified.

### Hospitalizations and Deaths

Among all dengue cases reported during 2010–2024, a total of 15,077 (38.6%) patients were hospitalized (Table 2). The highest rates of hospitalization were among patients with DENV-3 (60.9%) identified, followed by those with DENV-2 (50.2%); the lowest rates were among patients with DENV-1 (37.3%). Higher dengue hospitalization rates were also reported among patients aged <20 years (42.8%) than among those aged 20–39 (33.0%) and ≥40 years (36.3%). Just over one third of patients with dengue reported during 2010–2019 and 2020–2022 were hospitalized (35.7% and 38.0%, respectively); during 2023–2024 more than one half (53.5%) of patients with dengue were hospitalized. Among patients aged <20 years, hospitalization rates increased from 40.3% during 2010–2019 to 59.0% during 2023–2024. Similarly, among patients aged 20–39 years, hospitalization rates increased from 29.7% during 2010–2019 to 47.7% during 2023–2024. Among patients aged ≥40 years, approximately one third (32.8%) were hospitalized during 2010–2019; this percentage decreased to 28.4% during 2020–2022, but then increased to 52.1% during 2023–2024.

**TABLE 2:** Age group, pregnancy status, and infecting serotype among all dengue patients and hospitalized dengue patients — Puerto Rico Department of Health, Puerto Rico, 2010–2019, 2020–2022, and 2023–2024

Characteristic	2010–2019*		2020–2022*			2023–2024*			Total 2010–2024*		
	No. of dengue cases	No. (%) of hospitalized dengue patients	No. of dengue cases	No. (%) of hospitalized dengue patients	Adjusted* OR (95% CI)	No. of dengue cases	No. (%) of hospitalized dengue patients	Adjusted* OR (95% CI)	Total no. of dengue cases	No. (%) of hospitalized dengue patients	Adjusted* OR (95% CI)
Age group, yrs^†^											
<20	15,508	6,254 (40.3)	1,490	658 (44.2)	1.2 (1.0–1.3)	2,255	1,330 (59.0)	1.5 (1.3–1.7)^§^	**19,253**	**8,242 (42.8)**	**1.6 (1.5–1.7)^§^**
20–39	7,142	2,121 (29.7)	692	222 (32.1)	1.1 (1.0–1.3)	1,681	801 (47.7)	1.8 (1.6–2.1)^§^	**9,515**	**3,144 (33.0)**	**Ref**
≥40	7,489	2,457 (32.8)	511	145 (28.4)	0.8 (0.6–0.9)^§^	1,946	1,013 (52.1)	1.8 (1.6–2.0)^§^	**9,946**	**3,615 (36.3)**	**1.2 (1.1–1.2)^§^**
Unknown	378	76 (20.1)	2	0 (—)	—	0	0 (—)	—	**380**	**76 (20.0)**	**—**
**Total**	**30,517**	**10,908 (35.7)**	**2,695**	**1,025 (38.0)**	**1.1 (1.0–1.2)**	**5,882**	**3,144 (53.5)**	**1.7 (1.5–1.8)** ^§^	**39,094**	**15,077 (38.6)**	**—**
**Pregnant patients**	276	103 (37.3)	20	6 (30.0)	—	22	8 (36.4)	—	**318**	**117 (36.8)**	**—**
**Serotype^†^**											
DENV-1	15,039	5,360 (35.6)	1,404	521 (37.1)	1.1 (1.0–1.2)	1,629	855 (52.5)	2.2 (2.0–2.4)^§^	**18,072**	**6,736 (37.3)**	**Ref**
DENV-2	635	173 (27.2)	89	37 (41.6)	1.9 (1.2–3.0)^§^	940	626 (66.6)	5.3 (4.3–6.7)^§^	**1,664**	**836 (50.2)**	**1.5 (1.3–1.7)^§^**
DENV-3	5	1 (20.0)	18	9 (50.0)	4.2 (0.5–93.0)	2,183	1,333 (61.1)	6.6 (1.0–130.6)	**2,206**	**1,343 (60.9)**	**1.8 (1.6–2.0)^§^**
DENV-4	5,104	1,743 (34.1)	1	1 (100.0)	—	1	0 (—)	—	**5,106**	**1,744 (34.2)**	**1.0 (0.9–1.0)**
Unknown	9,734	3,631 (37.3)	1,183	457 (38.6)	—	1,129	330 (29.2)	—	**12,046**	**4,418 (36.7)**	**—**
**Total**	**30,517**	**10,908 (35.7)**	**2,695**	**1,025 (38.0)**	**1.1 (1.0–1.2)**	**5,882**	**3,144 (53.5)**	**1.7 (1.5–1.8)** ^§^	**39,094**	**15,077 (38.6)**	**—**

**Abbreviations**: DENV = dengue virus; OR = odds ratio; Ref = referent group.

* Each row in the first three columns (2010–2019, 2020–2022, and 2023–2024) represents the number and percentage of hospitalizations among cases in each age group or serotype, with adjusted ORs comparing proportions in 2020–2022 and 2023–2024 with those from 2010–2019 (Ref ). Age group ORs are adjusted by serotype. Serotype ORs are adjusted by age group. The “Total (2010–2024)” column represents a separate analysis comparing all hospitalized dengue patients during 2010–2024 with Ref categories for age group (20–39 years) and serotype (DENV-1).

^†^ Percentages of overall hospitalized dengue patients are calculated among those for whom information on age and DENV serotype were known.

^§^ Statistically significant increase or decrease in odds of hospitalization compared with 2010–2019.

During 2010–2024, a total of 318 (0.8%) of 39,094 dengue cases occurred in pregnant persons, including 276 (0.7%) during 2010–2019, 20 (0.1%), during 2020–2022, and 22 (0.1%) during 2023–2024. Hospitalization rates among pregnant patients with dengue during these periods were 37.3%, 30.0%, and 36.4%, respectively.

Hospitalization rates among patients infected with DENV-1 increased from 35.6% during 2010–2019 to 52.5% during 2023–2024. Similarly, among patients infected with DENV-2, hospitalization rates increased from 27.2% during 2010–2019 to 41.6% during 2020–2022, and to 66.6% during 2023–2024. The small numbers of DENV-3 and DENV-4 cases limited analyses of hospitalization rates over time for patients infected with these serotypes.

The percentage of fatal dengue cases did not change significantly over time. The highest number of fatal cases (77; 0.3%) was reported during 2010–2019; eight (0.3%), and nine (0.2%) fatalities occurred during 2020–2022 and 2023–2024, respectively.

## Discussion

These findings revealed a shift in the age distribution and percentage of hospitalized dengue patients during the analysis period, with an increase in the median patient age during the previous 2 years compared with both 2010–2019 and 2020–2022. This shift is likely the result of changing levels of population immunity, with lower levels of transmission during 2014–2019 after the large 2010–2013 outbreaks, resulting in lower levels of immunity among adults, driving a shift to slightly older age groups. In previous years, frequent exposure resulted in higher levels of protective immunity against DENV among adults. A similar trend has been reported in other countries, including Bangladesh, Indonesia, Singapore, and Thailand, where the average age of patients with dengue has increased over time (*4*,*5*).

During 2023–2024, DENV-3 gained predominance in Puerto Rico. The lack of previous population exposure to DENV-2 and DENV-3 might be associated with higher infection and transmission rates, increasing the risk for an outbreak. The emergence of DENV-3 has resulted in multiple large outbreaks in recent years. In Brazil, DENV-3 reemerged in 2023, 15 years after the last DENV-3 outbreak, resulting in an unprecedented DENV-3 outbreak in 2024 (*6*). In Cuba, DENV-3 resulted in a large outbreak in 2022 (*7*), and in Mexico, increased numbers of cases attributed to DENV-3 occurred in 2024 (*8*).

Several factors likely contributed to the increased proportion of hospitalized patients during 2023–2024 in Puerto Rico. DENV-2 and DENV-3 have been associated with higher rates of severe disease and hospitalization compared with DENV-1 (*9*). However, PRDH also strengthened surveillance by expanding dengue testing in commercial laboratories and initiating interviews with all patients in 2023–2024, resulting in more complete case ascertainment. Population immunity could also affect disease severity; longer intervals between DENV infections have been associated with increased disease severity (*10*). The increase in median patient age could also play a role, because older populations might experience higher hospitalization rates owing to the presence of underlying comorbidities. Changes might also be related to health care provider testing and reporting practices if more severe cases have been more likely to be reported in recent years.

### Limitations

The findings in this report are subject to at least five limitations. First, the associations evaluated across periods were not adjusted for confounders, such as comorbidities; patients with comorbidities might have higher hospitalization risk. Second, reported dengue cases do not include illnesses among persons who did not receive testing or did not seek health care services, likely underestimating the number of dengue cases in the community. Third, reporting biases could affect observed trends; hospitalized patients might have been more likely to be reported to public health authorities, inflating the proportion of hospitalized patients with dengue. Fourth, hospitalization practices might have changed over time; however, CDC recommendations** for hospitalization of persons with dengue have not changed throughout the analysis period. Finally, increases in the percentage of older adults in Puerto Rico might affect the proportion of cases observed in older age groups; however, dengue incidence increased among persons aged ≥20 years during 2023–2024 compared with 2010–2019.

### Implications for Public Health Practice

Reemerging DENV serotypes and lowered population immunity could increase the risk for dengue-related hospitalizations in Puerto Rico. Hospitalization rates were higher among patients infected with DENV-2 and DENV-3, which could result in higher numbers of hospitalizations if these serotypes continue to predominate in Puerto Rico. Public health partners can increase health care system preparedness for dengue. For example, efforts to accelerate the development and licensing of dengue vaccines for all age groups could be an important prevention tool. Additional education for health care providers could also strengthen dengue responses. During 2023–2024, just over one third (36.4%) of pregnant persons with dengue were hospitalized, although all pregnant persons with suspected dengue should be hospitalized or under close observation because of the risk for progression to severe dengue. Finally, raising awareness among health care providers and the public about the changing epidemiology of dengue could facilitate early detection and prompt management of cases.

## Footnotes

*
https://iris.who.int/bitstream/handle/10665/75303/9789241504034_eng.pdf

†
https://www.fda.gov/media/124379/download

§
https://ndc.services.cdc.gov/case-definitions/dengue-virus-infections-2015/

¶ 45 C.F.R. part 46.102(l)(2), 21 C.F.R. part 56; 42 U.S.C. Sect. 241(d); 5 U.S.C. Sect. 552a; 44 U.S.C. Sect. 3501 et seq.

**
https://www.cdc.gov/dengue/hcp/pocketguide/index.html
